# Association of Human Herpesvirus-6B with Mesial Temporal Lobe Epilepsy

**DOI:** 10.1371/journal.pmed.0040180

**Published:** 2007-05-29

**Authors:** Julie Fotheringham, Donatella Donati, Nahid Akhyani, Anna Fogdell-Hahn, Alexander Vortmeyer, John D Heiss, Elizabeth Williams, Steven Weinstein, Derek A Bruce, William D Gaillard, Susumu Sato, William H Theodore, Steven Jacobson

**Affiliations:** 1 Viral Immunology Section, National Institute of Neurological Disorders and Stroke, National Institutes of Health, Bethesda, Maryland, United States of America; 2 Struttura Complessa di Microbiologia e Virologia, Azienda Ospedaliera Universitaria Senese, Siena, Italy; 3 Department of Clinical Neuroscience, Division of Neurology, Karolinska Institutet, Stockholm, Sweden; 4 Surgical Neurology Branch, National Institute of Neurological Disorders and Stroke, National Institutes of Health, Bethesda, Maryland, United States of America; 5 Children's National Medical Center, Washington, District of Columbia, United States of America; 6 Clinical Epilepsy Section, National Institute of Neurological Disorders and Stroke, National Institutes of Health, Maryland, United States of America; Imperial College London, United Kingdom

## Abstract

**Background:**

Human herpesvirus-6 (HHV-6) is a β-herpesvirus with 90% seroprevalence that infects and establishes latency in the central nervous system. Two HHV-6 variants are known: HHV-6A and HHV-6B. Active infection or reactivation of HHV-6 in the brain is associated with neurological disorders, including epilepsy, encephalitis, and multiple sclerosis. In a preliminary study, we found HHV-6B DNA in resected brain tissue from patients with mesial temporal lobe epilepsy (MTLE) and have localized viral antigen to glial fibrillary acidic protein (GFAP)–positive glia in the same brain sections. We sought, first, to determine the extent of HHV-6 infection in brain material resected from MTLE and non-MTLE patients; and second, to establish in vitro primary astrocyte cultures from freshly resected brain material and determine expression of glutamate transporters.

**Methods and Findings:**

HHV-6B infection in astrocytes and brain specimens was investigated in resected brain material from MTLE and non-MTLE patients using PCR and immunofluorescence. HHV-6B viral DNA was detected by TaqMan PCR in brain resections from 11 of 16 (69%) additional patients with MTLE and from zero of seven (0%) additional patients without MTLE. All brain regions that tested positive by HHV-6B variant-specific TaqMan PCR were positive for viral DNA by nested PCR. Primary astrocytes were isolated and cultured from seven epilepsy brain resections and astrocyte purity was defined by GFAP reactivity. HHV-6 gp116/54/64 antigen was detected in primary cultured GFAP-positive astrocytes from resected tissue that was HHV-6 DNA positive—the first demonstration of an ex vivo HHV-6–infected astrocyte culture isolated from HHV-6–positive brain material. Previous work has shown that MTLE is related to glutamate transporter dysfunction. We infected astrocyte cultures in vitro with HHV-6 and found a marked decrease in glutamate transporter EAAT-2 expression.

**Conclusions:**

Overall, we have now detected HHV-6B in 15 of 24 patients with mesial temporal sclerosis/MTLE, in contrast to zero of 14 with other syndromes. Our results suggest a potential etiology and pathogenic mechanism for MTLE.

## Abstract 2

Steve Jacobson and colleagues report finding human herpesvirus-6B DNA in brain resections from 11 of 16 patients with mesial temporal lobe epilepsy, strengthening the evidence for a role for this virus in this condition.

## Editors' Summary

**Background.:**

Epilepsy is a common brain disorder caused by a sudden, excessive electrical discharge in a cluster of neurons—the cells that transmit electrical messages between the body and the brain. Its symptoms depend on which part of the brain is affected by this electrical firestorm and how far the disturbance spreads. When only part of the brain is affected (a partial seizure or fit), patients may see or smell strange things, recall forgotten memories, or have part of their body jerk uncontrollably. When the electrical disturbance spreads across the whole brain (a generalized seizure), there may be loss of consciousness and/or the whole body may become rigid or jerk. Epilepsy is usually controlled with anti-epileptic drugs or, in very severe focal cases, surgery to the area of the brain where the seizure starts. Although head injuries or brain tumors can trigger epilepsy, the cause of most cases of epilepsy is unknown.

**Why Was This Study Done?:**

Knowing what causes epilepsy might lead to better treatments for it. One possibility is that infections trigger epilepsy. The researchers in this study asked whether infections with human herpesvirus 6B (HHV-6B) are associated with a common type of epilepsy called mesial temporal lobe epilepsy (MTLE). Patients with MTLE often have extensive scarring in the hippocampus, a brain region responsible for memory that lies deep within a bigger region called the temporal lobe. Hippocampal scarring and MTLE are associated with a history of fever-induced fits, and HHV-6B infection can cause such fits in young children. Most people become infected with HHV-6B (or the closely related HHV-6A) early in life. The virus then remains latent for years within the brain and elsewhere. Given these facts and a previous investigation that showed that brain tissue from several patients with MTLE contained HHV-6B, the researchers reasoned that it was worth investigating HHV-6B as a cause of MTLE.

**What Did the Researchers Do and Find?:**

The researchers first looked for HHV-6B DNA in brain tissue surgically removed from patients with MTLE or another type of epilepsy. Tissue from 11 of 16 patients with MTLE (but from 0 of 7 control patients) contained HHV-6B DNA. When the researchers grew astrocytes (a type of brain cell) from some of these samples, only those from HHV-6B DNA-positive samples from patients with MTLE expressed an HHV-6-specific protein. Next, the researchers investigated in detail a patient with MTLE who had four sequential operations to control his epilepsy. This patient's hippocampus, which was removed in his first operation, contained a higher level of HHV-6B DNA than the tissues removed in later operations. After the fourth operation (which removed half of his brain and cured his epilepsy), astrocytes grown from the temporal lobe and the frontal/parietal lobe (a brain region next to the temporal lobe) but not the frontal and occipital lobes contained HHV-6B DNA and expressed a viral protein. The researchers also measured the production by these various astrocytes of a substance that moves glutamate (an amino acid that also acts as a neurotransmitter) across cell membranes—MTLE has been associated with a glutamate transporter deficiency. Consistent with this, astrocytes from the patient's temporal lobe made no glutamate transporter mRNA (mRNA is an essential precursor for protein to be produced). Finally, infection of astrocytes isolated from a patient without MTLE with HHV-6B greatly reduced expression of glutamate transporter in these astrocytes.

**What Do These Findings Mean?:**

These findings, together with those from the previous study, reveal that nearly two-thirds of patients with MTLE (but no patients with other forms of epilepsy) have an active HHV-6B infection in the brain region where their epilepsy originates. Overall, they provide strong support for the idea that HHV-6B infections might cause MTLE, particularly given the results obtained from the patient whose condition only improved after multiple brain operations had removed all the virally infected material. Furthermore, the demonstration that HHV-6B infection reduces glutamate transporter expression in astrocytes suggests that HHV-6B infection might cause astrocyte dysfunction. This dysfunction could lead to injury of the sensitive neurons in the hippocampus and trigger MTLE. Additional patients now need to be studied both to confirm the association between HHV-6B infection and MTLE and to discover exactly how this virus triggers epilepsy.

**Additional Information.:**

Please access these Web sites via the online version of this summary at http://dx.doi.org/10.1371/journal.pmed.0040180.

- MedlinePlus encyclopedia page on epilepsy (in English and Spanish)
- World Health Organization fact sheet on epilepsy (in English, French, Spanish, Russian, Arabic, and Chinese)
- US National Institute for Neurological Disorders and Stroke epilepsy information page (in English and Spanish)
- UK National Health Service Direct information for patients on epilepsy (in several languages)
- Neuroscience for kids, an educational Web site prepared by Eric Chudler (University of Washington, Seattle, Washington, United States), who also has a site that includes information on epilepsy and a list of links to epilepsy organizations (mainly in English but some sections in other languages as well)
- A short scientific article on human herpes virus 6 in the journal *Emerging Infectious Diseases*

## Introduction

Human herpesvirus-6 (HHV-6) is a β-herpesvirus first isolated in 1986 from immunosuppressed patients with lymphoproliferative disorders and HIV infection [1]. HHV-6 infects most humans between 6 and 12 mo of age [2], and more than 90% of the general population is seropositive [3]. After primary infection, HHV-6 can establish lifelong latency, with the viral genome persisting in peripheral blood mononuclear cells (PBMCs), salivary glands [4], and the central nervous system (CNS) [5]. HHV-6 DNA has been detected in the cerebrospinal fluid of children during primary infection and subsequent to infection, indicating CNS viral persistence [6]. HHV-6 reactivation may contribute to disease in immunosuppressed patients following bone marrow or solid-organ transplantation, and in those with chronic fatigue syndrome [7,8].

Two variants of the virus have been identified, A and B, with nucleotide sequence homology between 88% and 96%. HHV-6A has been implicated in multiple sclerosis, and is associated with viral persistence and reactivation in the CNS [9,10]. HHV-6B is primarily associated with symptomatic infections during infancy and is the causative agent of exanthem subitum. Although HHV-6B is detected more frequently in PBMCs of healthy adults in the US population and may constitute the majority of latent HHV-6 infection, HHV-6A may be more neurotropic [11–13].

While associations with adult neurologic disease [14–17] are thought to involve reactivation of latent HHV-6, primary HHV-6 infection is associated with febrile seizures in both infants and young children. The incidence of febrile seizures with primary HHV-6 infection ranges from 8%–20% in the US population [18–20].

Epilepsy is one of the most common and severe neurological disorders, with a prevalence of 0.6%–1%, and a large social and economic cost [21]. Despite recent diagnostic advances, the etiology of epilepsy in a high percentage of patients remains unknown, although a wide range of infections is associated with the broad category of seizure disorders. Mesial temporal lobe epilepsy (MTLE) is one of the most common and intractable forms of seizure disorder. MTLE usually begins in childhood and is often associated with a history of prolonged or complex (and possibly simple) febrile seizures [22,23]. Mesial temporal sclerosis (MTS), with extensive astrogliosis and neuronal loss, is the most common pathological finding in MTLE. Imaging studies have found an association between febrile seizures and MTS [24,25].

Temporal lobectomy is an effective treatment for MTLE, achieving significantly better seizure control than antiepileptic drugs [26]. In a preliminary study of brain resections from patients with MTLE, we detected HHV-6B DNA at high viral loads in hippocampal sections, with viral antigen colocalized to glial fibrillary acidic protein (GFAP)–positive cells, confirming the in vivo localization of HHV-6 to astrocytes [15]. HHV-6 is present in astrocytes in brain tissue from patients with multiple sclerosis [27], limbic encephalitis [13], and post–bone marrow transplantation encephalitis, suggesting that astrocytes may be an in vivo reservoir for HHV-6. In vitro, HHV-6 infects a wide variety of cell types, including primary human astrocytes [28–31].

Here, we extend our previous investigations on the prevalence of HHV-6 infection in the hippocampus and temporal lobe from a new series of patients with MTLE and non-MTLE–related intractable epilepsy.

## Materials and Methods

### Patients

We studied 22 new patients (Table 1) referred for evaluation to the Clinical Epilepsy Section, National Institute of Neurological Disorders and Stroke, National Institutes of Health (NINDS/NIH; Bethesda, Maryland), or to the Department of Neurology Children's National Medical Center (CNMC; Washington, D. C.). Of these, 13 had surgery at NIH and nine had surgery at CNMC. Protocols for patient evaluation and surgical sample collection were approved by the NINDS and CNMC Intramural Clinical Research Committees. Clinical evaluation in each case included ictal video-EEG monitoring, 3-D–volumetric fast spin echo; and axial T1 and T2, coronal T2, and flair sequences performed on a 1.5 Tesla scanner (General Electric, http://www.gehealthcare.com). Mean age at seizure onset was 10.8 ± 10.7 y, and mean age at surgery was 21.4 ± 12.4 y. The 15 patients with MTLE had a significantly older age at surgery (27.4 y versus 14.9 y; *p* < 0.03) and a nonsignificant trend toward lower seizure onset age (7 y versus 12 y) than the seven patients without MTLE. Five patients with MTLE, but no patients without MTLE, had a history of febrile seizures (Table 1). All the patients with MTLE, compared with only one patient without MTLE who had a ganglioglioma, had clinical, electrographic, and/or imaging characteristics of MTLE [32,33]. We also studied one additional patient (patient 16) who presented with typical temporal lobe epilepsy but then developed a diffuse epileptic syndrome, resulting in a hemispherectomy.

**Table 1:**
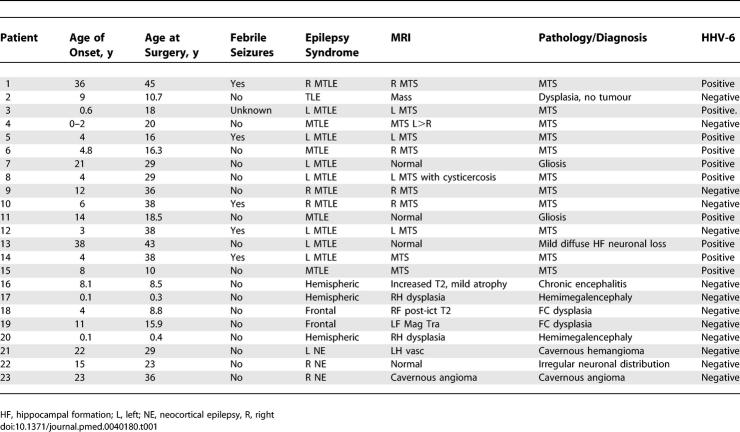
Clinical Information from NIH/CNMC Epilepsy Patient Cohort

The patient 16 was an 8-y-old, right-handed boy with a history of febrile seizures presented with sudden onset of frequent complex partial and rare secondary generalized seizures, without clear etiology. Complex partial seizures were characterized were characterized by anxiety and repetition of a short verbal phrase, followed by staring and aphemia with preserved comprehension. Initial EEG showed left-sided slowing; MRI showed left temporal fullness and increased T2 signal. Cerebrospinal fluid revealed no red and three white cells, a protein of 13, and glucose of 77 mg/100 ml. Despite negative PCR for herpes, acyclovir was started. Seizures persisted despite eight antiepileptic drugs, steroids, IVIG, ganciclovir, and a vagal nerve stimulator. EEG showed left temporal periodic epileptiform discharges and left frontal and right sharp waves. A left mesial temporal lobectomy led to transient improvement, but within 2 mo seizure frequency approached baseline levels with semiology unchanged. MRI was unchanged, and there were no focal neurological findings. Invasive monitoring revealed left mid/posterior superior temporal gyrus seizure onset. After resection, frequent electrographic and clinical seizures arose from inferior and middle temporal gyrus and frontal lobe. After several additional focal resections, a hemispherectomy was performed. The patient has been seizure free without anticonvulsants for 1 y. He has a right hemiparesis with little finger function, mild receptive deficits, and more severe expressive deficits; language is improving.

All patient surgical samples were reviewed by a clinical neuropathologist before submission for virological study. Samples from mesial temporal and neocortical resections and lateral temporal lobe resections were fixed in formalin and processed for standard pathological examination. All patients with MTS/MTLE showed variable degrees of neuronal loss and gliosis preferentially affecting the CA1 and CA3 areas, but also markedly affecting CA4, CA2, and the dentate gyrus in more severe cases. There was no evidence of inflammation or neuronal inclusions in any patient with MTS/MTLE.

### Sample Collection

For isolation of PBMCs, blood samples were drawn into acid citrate dextrose solution A tubes (Becton Dickinson, http://www.bd.com) and were separated using lymphocyte separation medium (ICN Biomedicals, http://www.mpbio.com). Cells were stored in liquid nitrogen prior to DNA extraction. Serum samples were extracted within 4 h of collection. Fresh brain material was obtained during epilepsy brain resection. Brain tissue was stored on ice in Hibernate A medium, and astrocyte isolation was initiated within 30 min after obtaining tissue.

### DNA and RNA Extraction

DNA and RNA were isolated from fresh brain tissue using commercially available extraction kits according to manufacturers' instructions. The QIAamp blood kit was used for DNA extraction from PBMCs, the QIAamp Viral RNA kit was used for DNA extraction from serum (1.2 ml), and the DNeasy tissue kit (all kits from Qiagen, http://www.qiagen.com) was used for DNA extraction from fresh brain tissue. DNA and RNA were also extracted from cultured primary astroytes obtained from patient brain resections using DNeasy and RNeasy extraction kits (Qiagen). For RNA extraction from tissue, finely minced brain samples were resuspended in 350 μl QIAzol lysis reagent (Qiagen) and stored at −70 °C until use. RNA extraction was performed as per the manufacturer's directions using the RNeasy lipid tissue mini kit (Qiagen).

### Nested PCR for HHV-6 Major Capsid Protein

DNA amplification was performed, using nested primers specific for a highly conserved sequence corresponding to the *major capsid protein* gene *(MCP)* of HHV-6 [9,34]. The external primers amplified a 520 bp sequence, and the internal primers amplified a 258 bp sequence. PCR was performed using the Taq PCR master mix kit (Qiagen) as per manufacturer's instructions. DNA was amplified with 0.5 μM final primer concentration for 35 cycles using the following conditions: denaturation at 92 °C for 0.3 min, annealing at 55 °C for 0.3 min, and extension at 72 °C for 0.32 min. A total of 5 μl of primary PCR product was amplified using the internal primers with the same PCR conditions. A total of 10 μl of PCR product was subjected to electrophoresis on a 1.5% agarose gel and visualized by ethidium bromide staining.

### Real-Time Quantitative PCR

Viral DNA in patient samples was quantified using TaqMan PCR with primers specific for HHV-6A and HHV-6B as described previously [15,35]. The A- and B-specific primers are located within the immediate early region of HHV-6 and bind specifically to their respective variants. Control experiments demonstrated that A-specific primers amplified only the HHV-6A laboratory strains U1102 and GS, and that B-specific primers amplified the HHV-6B laboratory strain Z29 [15]. Standard and sample DNA were amplified in a 96-well reaction plate using the following conditions: 50 °C for 2 min for activation of uracil-*N*-glycosylase, 95 °C for 10 min to inactivate uracil-*N*-glycosylase, and 45 cycles of 95 °C for 15 s (denaturation) and 60 °C for 1 min (annealing and extension). All standards and samples were assayed in triplicate, and HHV-6 viral load was normalized to actin.

### Primary Astrocyte Culture

Fresh tissue was obtained from epilepsy brain resections performed at NIH or CNMC. Tissue was kept on ice in Hibernate A medium (containing 2% B27 supplement, 0.5 mM glutamine, fungicide, and 1% penicillin/streptomycin). Meninges and blood vessels were removed, and tissue was minced into small pieces and dissociated in Earl balanced salt solution (containing 0.01% DNase, 20 U/ml papain, and 1:100 penicillin/streptomycin) in a 37 °C shaking water bath for 1 h. Digested tissue was mechanically dissociated with sterile pipettes and passed through a 60 μm filter. The single-cell suspension was centrifuged at 1,500 rpm for 10 min and separated on a Percoll gradient for 30 min at 15,000 rpm. The glial cell layer was isolated, washed, and plated overnight in poly-L-lysine coated flasks or two-well chamber slides in astrocytic medium (DMEM/F12 containing 10% FBS, 1% penicillin/streptomycin, and 0.01% gentamicin). Primary cultures were allowed to adhere overnight, and fresh medium was added the following day. After 3–4 wk in culture, astrocytes were stained for GFAP to determine culture purity.

### Immunofluorescence

Primary astrocytes were isolated from patients with MTLE included in Table 2. Astrocytes from epilepsy patients 15 (Table 1) and patient 2a (previously described as patient 2 [15]) were grown on poly-L-lysine–coated chamber slides for 3–4 wk. Primary antibodies were prepared in PBS and consisted of 1:100 mouse anti-gp116/54/64 (Advanced Biotechnologies, http://www.abionline.com), 1:100 rabbit anti-GFAP (DAKO, http://www.dako.com), and 1:50 mouse anti-microglia marker CD68 (Santa Cruz Biotechnology, http://www.scbt.com). Slides were incubated with primary antibodies for 1 h at room temperature and then washed three times in PBS. Secondary antibodies conjugated to the appropriate fluorophore (Molecular Probes, http://probes.invitrogen.com) consisted of 1:100 anti-rabbit IgG FITC (green), 1:1,000 anti-mouse IgG rhodamine (red), and 1:100 anti-mouse IgG AMCA (blue). Slides were incubated with secondary antibody for 1 h at room temperature and washed three times in PBS. Where indicated, slides were counterstained with DAPI in mounting media (Molecular Probes). Mounted slides were visualized using a fluorescence microscope (Carl Zeiss, http://www.zeiss.com) at 20×, 32×, or 40× magnification.

**Table 2:**
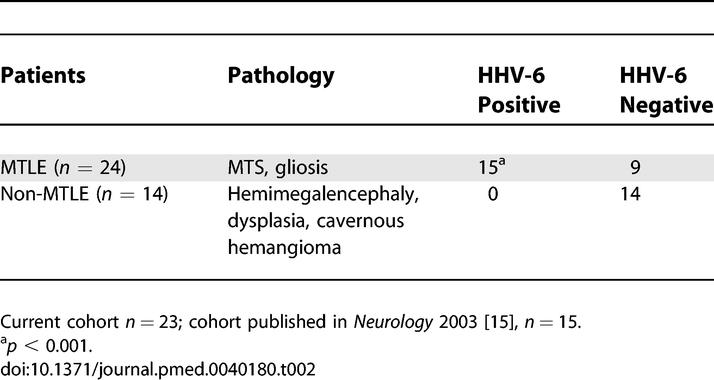
HHV-6 DNA Detection in Temporal Lobe Resections from NIH/CNMC epilepsy cohort (n=38)

### HHV-6 Infection of Primary Astrocytes

After 2–3 wk in culture, primary astrocytic cells from patient 20 were infected with HHV-6A and HHV-6B. Infection was performed as described previously [28]. Briefly, cultures were infected with freshly thawed cell-free supernatant from HHV-6A–infected JJahn or HHV-6B–infected SupT-1 cells at a ratio of 10^3^ DNA viral copies/cell (quantified by TaqMan PCR). Mock infections were performed using culture medium from uninfected SupT1 or JJahn. Cultures were incubated for 3 h at 37 °C in 5% CO_2_, cultures were washed three times with PBS, and fresh medium was added. Cells were harvested for experiments 5 d after infection.

### Reverse Transcription-PCR and TaqMan for EAAT-2

RNA extracted from primary astrocytes was reverse transcribed using reagents from Applied Biosystems (http://www.appliedbiosystems.com) according to manufacturer's instructions. cDNA was amplified using primers specific for EAAT-2 [36]. PCR for EAAT-2 was run for 35 cycles using the following conditions: 95 °C for 45 s, 55 °C for 1 min, and 72 °C for 1 min. cDNA was also amplified using TaqMan primer/probe sequences specific for EAAT-2 and HPRT (synthesized from cDNA sequences by Synthegen, now Integrated DNA Technologies, http://www.idtdna.com). cDNA was amplified in a 96-well reaction plate using the following conditions: 50 °C for 2 min for activation of uracil-*N*-glycosylase, 95 °C for 10 min to inactivate uracil-*N*-glycosylase, and 45 cycles of 95 °C for 15 s (denaturation) and 60 °C for 1 min (annealing and extension).

## Results

### Quantitative Detection of HHV-6B DNA in Brain Tissue

**Figure 1:**
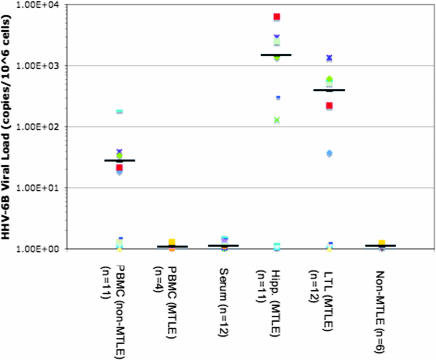
High Levels of HHV-6 DNA Are Detected in Brain Resections from Patients with MTLE HHV-6B DNA was quantitated in PBMCs, serum, and fresh brain (hipp, hippocampus; LTL, lateral temporal lobe) from epilepsy brain resections by TaqMan PCR. Viral load was normalized as DNA copies/10^6^ cells (brain and PBMCs) or DNA copies/ml (serum). Data from brain, serum, and PBMCs are represented graphically from patients with and without MTLE. Means are shown by black bars.

HHV-6 type–specific analysis amplified HHV-6B but not HHV-6A from a subset of patients with MTLE (Figure 1). The range of HHV-6 in normal PBMCs has been reported to be ≤100 copies/10^6^ cells [15], and no statistical difference was observed in quantitative HHV-6B levels in PBMCs isolated from patients with MTLE compared with those in patients without MTLE. In contrast, HHV-6B DNA levels were significantly higher in resected brain tissue from patients with MTLE compared with patients with epilepsy without MTLE (*p* < 0.001; Figure 1). Mean HHV-6B DNA levels in MTLE brain tissue (hippocampus and temporal lobe) were 1.5 × 10^3^ copies/10^6^ cells and 3.9 × 10^2^ copies/10^6^ cells respectively (Figure 1), while patients without MTLE had undetectable levels of HHV-6 DNA in brain resections. Notably, three hippocampus specimens resected from patients with MTLE, and four specimens from patients without MTLE were negative for HHV-6B DNA. The negative viral loads assessed by TaqMan were also confirmed by negative nested PCR findings.

### Characterization of HHV-6B Active Infection on Cultured Astrocytes from Patients with MTLE

**Figure 2:**
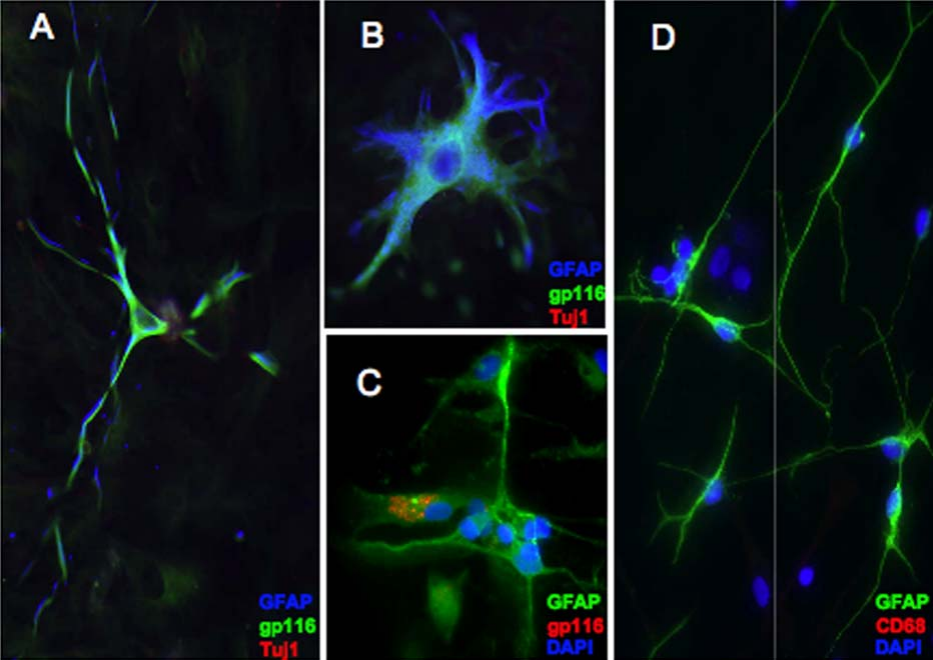
Primary Astrocytes Isolated and Cultured from HHV-6B–Positive MTLE Brain Resections Express Viral Antigen Primary astrocytes were isolated from fresh brain material obtained during epilepsy brain resection. Cells were cultured for 3–4 wk and costained for the nonvariant specific HHV-6 gp116 surface glycoprotein and GFAP as a marker for astrocytes (A–C), the neuronal marker Tuj1 (A–B), or the microglial marker CD68 (D). (A) Epilepsy patient 2a: GFAP = blue; HHV-6 = green; Tuj1 = red, 20×. (B) Epilepsy patient 2a: GFAP = blue; HHV-6 = green; Tuj1 = red, 32×. (C) Epilepsy patient 15; GFAP = green, HHV-6 = red; DAPI = blue, 40×. (D) Epilepsy patient 15; GFAP = green; CD68 = red; DAPI = blue, 40×.

From brain resections where sufficient quantities of tissue were available (five patients with MTLE and two patients without MTLE), primary glial cells were cultured and tested for purity of astrocyte populations by immunofluorescent-staining GFAP. Each culture demonstrated variable astrocyte purity by expression of GFAP (blue-stained cells in Figure 2A and 2B; green-stained cells in Figure 2C and 2D). Primary cultures were negative for the neuronal marker Tuj1 (β-tubulin III; lack of red staining in Figure 2A and 2B) and for the microglial marker CD68 (lack of red staining in Figure 2D), supporting the characterization of these cells as primary astrocytes. Culture conditions did not support growth of neurons or oligodendrocytes.

To determine whether primary astrocytes isolated from MTLE brain tissue contained HHV-6, cells were analyzed for the expression of the nonvariant specific HHV-6 surface glycoprotein gp116/54/64 by immunofluorescence. As shown in Figure 2, detection of HHV-6 gp116 in primary astrocytes from patients with MTLE demonstrated HHV-6 viral protein expression that colocalized with GFAP-positive astrocytic cells (green staining in Figure 2A and 2B; red staining in Figure 2C). The astrocytes in Figure 2 were cultured from the hippocampus/temporal lobe of MTLE brain resections, each of which was positive for HHV-6 by PCR and typed as HHV-6B by TaqMan (unpublished data). Examples of primary astrocytes from additional patients with MTLE expressing HHV-6 antigen and containing HHV-6 viral DNA is shown in Figure S1. By contrast, primary astrocyte cell lines cultured from HHV-6–negative patients without MTLE remained HHV-6 PCR negative and did not express HHV-6 gp116 by immunofluorescence (unpublished data).

### Longitudinal Detection and Characterization of HHV-6B

**Figure 3:**
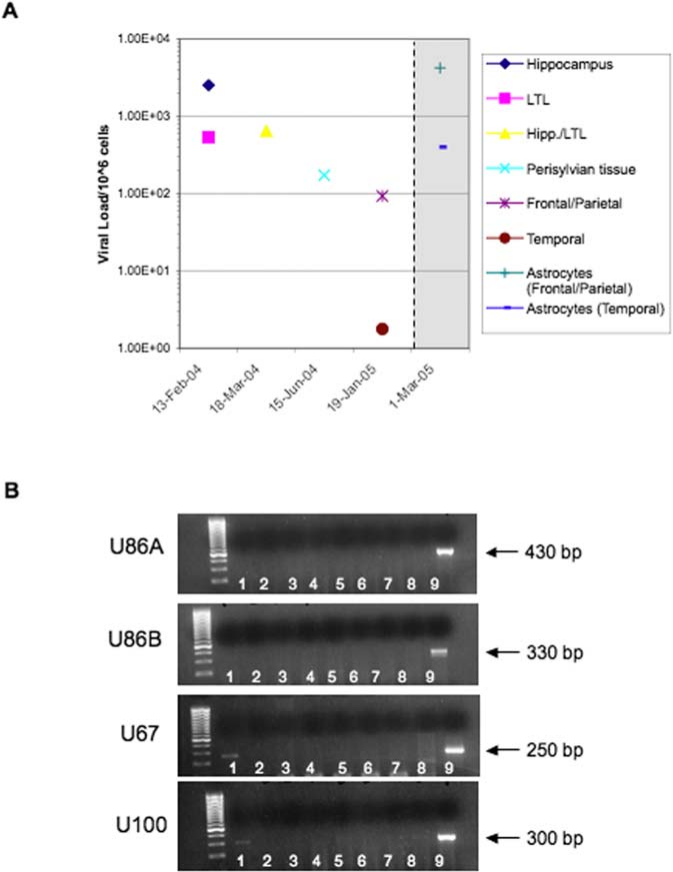
Longitudinal Characterization of HHV-6 Infection in One Patient with Multiple Brain Resections Followed by Hemispherectomy (A) HHV-6B viral load was quantitated from fresh tissue obtained from three consecutive brain resections (13 February 2004, 18 March 2004, and 15 June 2004) followed by hemispherectomy (19 January 2005) using variant-specific TaqMan PCR. Primary astrocytes were cultured from tissue obtained at hemispherectomy from frontal/parietal and temporal lobes. HHV-6B viral load was quantitated by Taqman in these astrocyte cultures (1 March 2005) approximately 6 wk after surgery. All viral loads are represented as DNA copies/10^6^ cells. (B) Expression of viral RNA was determined by RT-PCR using primers for three HHV-6 genes (from top to bottom: U86, immediate early [primers specific for variants A and B], U67, late; U100, late). Lane 1, patient 16 frontal/parietal lobe astrocytes; lane 2, patient 16 temporal lobe astrocytes; lane 3, negative control temporal lobe; lane 4, patient 16 frontal/parietal lobe; lane 5, patient 16 temporal lobe; lane 6, patient 16 occipital lobe; lane 7, patient 16 frontal lobe; lane 8, uninfected JJahn (negative control); lane 9, U1102-infected JJahn (positive control).

Between February 2004 and January 2005, patient 16 at CNMC had four brain resections for uncontrolled seizures, including a hemispherectomy in January 2005. Although patient 16 was not classified with classic MTLE, early symptoms indicated MTLE that progressed into a more severe syndrome eventually requiring hemispherectomy. In tissue from each brain resection, HHV-6 was detected by PCR (unpublished data) and was quantified and subtyped as HHV-6B by TaqMan PCR (Figure 3). The highest HHV-6 viral load was detected in the hippocampus following the first brain resection (2.53 × 10^3^/10^6^ cells; Figure 3). Sufficient quantity of brain material was obtained following hemispherectomy in January 2005 for isolation and culture of primary cells. Astrocyte cultures isolated from frontal/parietal and temporal lobes were HHV-6 gp116/54/64 positive and colocalized with GFAP (Figure 2A), indicating the presence of viral antigen in astrocytes in vitro. In addition, these cells were positive for HHV-6 DNA by PCR (unpublished data), and viral DNA was quantified and subtyped as HHV-6B (Figure 3). Notably, TaqMan HHV-6B viral DNA levels in these primary astrocyte cultures were higher than those quantified from DNA isolated from the corresponding regions of fresh brain, indicating the possibility of further viral reactivation during primary culture (Figure 3). DNA extracted from freshly resected temporal and frontal/parietal lobes following hemispherectomy was also positive for HHV-6 by PCR (unpublished data), and was quantified and typed as HHV-6B with viral loads of 1.77 copies/10^6^ cells and 93 copies/10^6^ cells, respectively. In contrast, DNA extracted from freshly resected frontal and occipital lobes was negative for HHV-6 by both PCR and TaqMan (unpublished data), indicating that viral infection was localized and not spread throughout the brain. In patients with MTLE, HHV-6 infection was localized primarily to the temporal lobe, with highest viral load detected in the hippocampus. Similarly, the first hippocampal resection of patient 16 demonstrated the highest viral load detected. Detection of HHV-6B DNA in the frontal/parietal lobe following hemispherectomy supports the finding that the syndrome progressed from early MTLE to a chronic encephalitis, and may have lead to spread of HHV-6 infection in the brain of this patient. Finally, we determined expression of HHV-6 RNA using a set of HHV-6–specific primers specific for HHV-6 *U67* and *U100* late genes. HHV-6 RNA was demonstrated in primary astrocytes isolated from the frontal/parietal lobe (Figure 3B), consistent with the detection of high levels of HHV-6B DNA (Figure 3A) and HHV-6 antigen by IFA (Figure 2A).

### Association of Active HHV-6B Infection and Impaired EAAT-2 mRNA Transcription in Cultured Astrocytes

**Figure 4:**
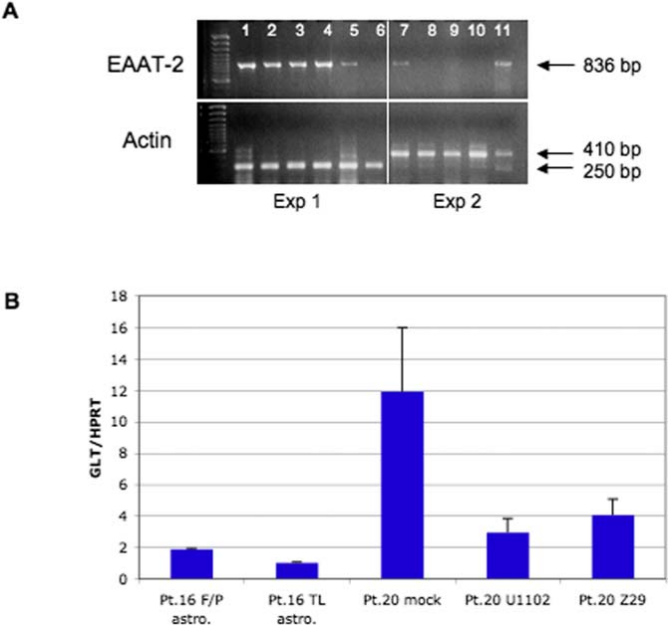
Decreased Expression of the Glutamate Transporter EAAT-2 in HHV-6–Infected Astrocytes (A) EAAT-2 mRNA was detected by RT-PCR in fresh brain from patient 16, in astrocytes cultured from patient 15, and in astrocytes cultured from patient 20, and infected with HHV-6 in vitro. Samples from the two different patients were run in two separate PCR reactions (on different days) using the same set of EAAT-2 primers but different actin primers. The products of these PCR reactions were run on the same gel. Lane 1, patient 16 frontal/parietal lobe; lane 2, patient 16 temporal lobe; lane 3, patient 16 occipital lobe; lane 4, patient 16 frontal lobe; lane 5, patient 16 frontal/parietal lobe astrocytes; lane 6, patient 16 temporal lobe astrocytes; lane 7, uninfected astrocytes cultured from patient 20; lane 8, HHV-6A (strain U1102)–infected astrocytes cultured from patient 20; lane 9, HHV-6B (strain Z29)–infected astrocytes cultured from patient 20; lane 10, negative control (JJhan T cells); lane 11, positive control (U251 astrocytes). (B) EAAT-2 mRNA was detected by quantitative TaqMan and normalized to expression of HPRT from astrocytes cultured from frontal/parietal (F/P) and temporal (TL) lobes of patient 16. Astrocytes from patient 20 were mock-infected or were infected with HHV-6A (strain U1102) or HHV-6B (strain Z29).

We measured transcription of the astrocytic glutamate transporter EAAT-2 mRNA in four brain regions and cultured primary astrocytes isolated from patient 16 by reverse transcription PCR (RT-PCR). All four brain regions expressed mRNA for EAAT-2, while primary astrocytes isolated from the frontal/parietal lobe expressed low levels of EAAT-2 and mRNA was undetectable in primary astrocytes isolated from the temporal lobe (Figure 4A). We compared mRNA expression of EAAT-2 in primary astrocytes isolated from patient 16 with primary astrocytes isolated from a patient who was HHV-6B DNA negative. Primary astrocytes from patient 20 (HHV-6B DNA negative) were also infected in vitro with HHV-6A and HHV-6B to compare EAAT-2 expression in astrocytes infected in vitro with astrocytes cultured from HHV-6–positive brain tissue. While EAAT-2 mRNA was expressed in mock-infected primary astrocytes from patient 20, EAAT-2 expression was dramatically reduced in primary astrocytes infected with either HHV-6A (U1102) or HHV-6B (Z29) (Figure 4A). These observations were confirmed by TaqMan, showing low levels of EAAT-2 expression in both primary astrocytes isolated from patient 16 and in the HHV-6A– and HHV-6B–infected primary astrocytes from patient 20 compared with the expression levels of EAAT-2 in mock-infected primary astrocytes (Figure 4B). Viral load in HHV-6A– and HHV-6B–infected primary astrocytes from patient 20 were 1.19 × 10^8^ copies/10^6^ cells and 1.68 × 10^6^ copies/10^6^ cells, respectively.

## Discussion

This study demonstrates persistent HHV-6B infection in most patients with MTS-MTLE, but no detectable infection in patients with other pathology and constitutes the first report of primary isolation and maintenance of virus-infected astrocytes from the human brain. In addition, we provide direct evidence for an etiological link of a ubiquitous human herpesvirus in a subset of patients with intractable MTLE with MTS, a syndrome of unknown origin. Active HHV-6 infection was confirmed by the detection of viral DNA, mRNA viral transcripts, and viral protein expression in human primary astrocytes isolated and cultured from resected brain tissue from patients with MTLE. HHV-6B in MTS-MTLE is unlikely to be a consequence of nonspecific inflammation or seizures, since none of the patients without MTLE patients, all of whom had intractable epilepsy, were positive for virus. Moreover, there was no evidence for inflammatory changes in any of the HHV-6B–positive patients.

Studies associating HHV-6 with neurologic disorders, including epilepsy, are based on detection of viral DNA, RNA, and antigen, suggesting pathogenic reactivation of latent HHV-6. Establishment of HHV-6 latency in the CNS can follow primary childhood infection [37,38]. Although HHV-6 reactivation in the brain appears to occur predominately in immunosuppressed adults and is associated with neurological complications following bone marrow and stem cell transplantation, viral reactivation may also play a role in disorders such as multiple sclerosis and epilepsy. Several studies have demonstrated the presence of low levels HHV-6 DNA (typically nested PCR) in normal human brains that can range between a frequency of 0%–75% [15,37–40]. These discrepancies can be attributed to different patient populations, methods of detection, and sensitivities and specificities of reagents. Studies demonstrating HHV-6 DNA in the normal human brain have typically used nested PCR, indicating that levels of viral DNA are low, supporting the observations by Blumberg et al. that HHV-6 may be present as a latent commensal pathogen that is able to infect the human brain without causing any apparent neurological disease [41]. The result in this study demonstrating high levels of viral DNA detected in MTLE specimens by non-nested TaqMan PCR, together with the presence of viral antigen and RNA, suggests association with disease. Detection of HHV-6 DNA by PCR in 25%–50% of patients with temporal lobe epilepsy indicates an association of viral reactivation with a neurological disorder that may be independent of immunosuppression [15,42–44]. The results in this study demonstrated higher HHV-6 levels in hippocampal tissue than in the surrounding temporal neocortex. High viral loads in the hippocampus from patients with MTLE could reflect viral reactivation in a specific brain region associated with latency resulting from early childhood infection.

Alternatively, persistent noninflammatory encephalitis may also lead to the development of epilepsy. Several lines of evidence, including clinical, imaging, and neuropsychological data, suggest that MTS/MTLE is a progressive disorder [45,46]. Imaging studies have shown that febrile seizure history and epilepsy duration are associated with increasing hippocampal atrophy, independent of seizure frequency [24,47,48]. A history of febrile seizures may be difficult to establish retrospectively, many years after the event. In our overall cohort of 38 patients, which represents our collective experience at NIH and the CNMC (Table 2), five of nine patients with a definite history of febrile seizures (all in the MTS-MTLE group), compared with nine of 28 reporting no history of febrile seizures, were positive for HHV-6B (a clear history could not be obtained for one HHV-6B–positive patient with MTS-MTLE), suggesting a trend towards HHV-6B–positive patients with MTLE having febrile seizure history. The long latency between childhood febrile seizures and the appearance of persistent unprovoked seizures suggests these patients may have chronic HHV-6 infection rather than reactivated virus. The presence of chronic viral infection in these patients would be supported by the progression of hippocampal atrophy. Collectively, these data suggest an ongoing process; the latency between occurrence of an early risk factor such as febrile seizures and onset of chronic epilepsy is consistent with either persistent or reactivated infection [25,32].

We also had the unique opportunity to follow longitudinally a patient who experienced recurrent seizures after three focal resections and a hemispherectomy for a period of 11 mo. Consistent detection of high levels of HHV-6B DNA in each of the three resections and in frontal and temporal/parietal lobes following hemispherectomy suggests this patient had a persistent and widespread infection with HHV-6B. Following hemispherectomy, this patient has been seizure-free without anticonvulsant treatment for more than 1 y. It is intriguing to speculate that these data demonstrate a widespread and persistent HHV-6 infection that was associated with epilepsy, and that once the viral infection was removed, the patient became seizure-free.

An association between chronic epilepsy and persistent or reactivated HHV-6 infection of astrocytes suggests the possibility that viral infection of astrocytes are associated with changes in cell function that may contribute to disease. Astrocytes are known to interact closely with neurons and are critical in modulating synaptic transmission [49]. Astrocytes can modulate neurotransmission by maintaining low concentrations of extracellular glutamate by the glial glutamate transporters EAAT-1 and EAAT-2 [50]. Elevated extracellular glutamate, the main excitatory neurotransmitter, may be involved in epilepsy by triggering excitotoxicity through loss of glutamine synthetase [51], an enzyme that metabolizes glutamate in astrocytes, and/or by malfunctioning astrocytic glutamate transporters [50]. The CA1 and CA3 neurons lost in MTS/MTLE are particularly susceptible to glutamatergic-mediated cell death. Sclerotic hippocampi from temporal lobe epilepsy demonstrate reduced EAAT-2 immunoreactivity [52], and are prone to alternative EAAT-2 mRNA splicing [53]. A unique finding in this study is the isolation ex vivo of cultured astrocytes from patients with MTLE who are infected with HHV-6. These primary HHV-6–infected astrocytes demonstrated low levels of EAAT-2 mRNA. In support of our ex vivo findings, astrocytes infected with HHV-6 in vitro also demonstrated a remarkable decrease in EAAT-2 mRNA. Detection of high levels of HHV-6 DNA in MTLE brain tissue, isolation of HHV-6 from primary astrocytes isolated from MTLE brain tissue, and decreased expression of EAAT-2 mRNA demonstrates an association between HHV-6 infection and astrocytic dysfunction. Functional changes in virus infected glia or in glia harboring reactivated virus may lead to secondary injury of the exquisitely sensitive hippocampal neuron, and ultimately to development of MTLE and epilepsy. The potential relationship between HHV-6 astrocytic infection and MTLE deserves further investigation.

Overall, our cumulative experience at the NIH Clinical Center and the CNMC consists of a large cohort of patients with epilepsy, from which 60% with clinically defined MTLE had detectable HHV-6B sequences in surgical brain resections. The highest HHV-6 viral loads were demonstrated in the hippocampus. This appeared to be specific for MTLE, since non-MTLE epilepsy material had no detectable levels of HHV-6 DNA by TaqMan or nested PCR. The statistically significant correlation of HHV-6 and MTLE (Table 2) in a large cohort of patients with epilepsy and dysregulation of glutamate transporter expression by HHV-6 suggests a novel pathophysiological mechanism of disease.

## Supporting Information

Figure S1 Primary Astrocytes Isolated and Cultured from HHV-6B–Positive MTLE Brain Resections Express Viral DNA and Antigen Primary astrocytes were isolated from fresh brain material obtained during epilepsy brain resection.
(A) Cells were cultured for 3–4 wk and stained for GFAP (green), DAPI (blue; nuclei), and the nonvariant specific HHV-6 antigen gp116/54/64 (red). Representative immunofluorescence images show primary astrocyte cultures from four epilepsy brain resections (patients 14, 15, 5, and 6). All images were acquired with a 20× objective.
(B) Cells were scraped from fixed slides (patients 5 and 6), DNA was extracted, and DNA for HHV-6 U57 (major capsid protein) was detected by nested PCR. Negative and positive controls used were uninfected SupT1 T cells and HHV-6B (strain Z29)–infected SupT1 T cells, respectively.
(36 KB JPG)

### Accession Numbers

The GenBank (http://www.ncbi.nlm.nih.gov/) accession numbers for the viruses and transporter discussed in this paper are HHV-6A (NC_001664), HHV-6B (NC_000898), and EAAT-2 (NM_004171).

## Abbreviations

CNS: central nervous system
GFAP: glial fibrillary acidic protein
HHV-6: human herpesvirus-6
MTLE: mesial temporal lobe epilepsy
MTS: mesial temporal sclerosis
PBMC: peripheral blood mononuclear cells
RT-PCR: reverse transcription PCR
