# Supplementary figures and images for: Association of Human Herpesvirus-6B with Mesial Temporal Lobe Epilepsy
Source: PLoS Med. 2007 May 29;4(5):e180. doi: 10.1371/journal.pmed.0040180 (PMC1880851; doi:10.1371/journal.pmed.0040180)

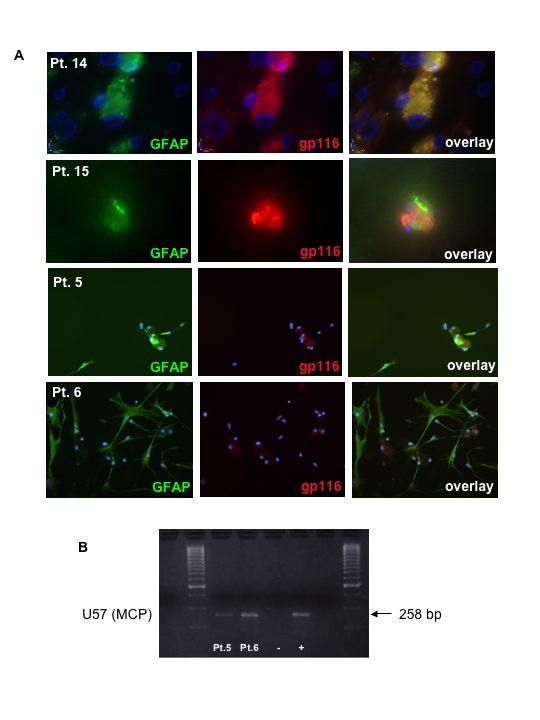

Supplement: Figure S1 — Primary astrocytes were isolated from fresh brain material obtained during epilepsy brain resection. (A) Cells were cultured for 3–4 wk and stained for GFAP (green), DAPI (blue; nuclei), and the nonvariant specific HHV-6 antigen gp116/54/64 (red). Representative immunofluorescence images show primary astrocyte cultures from four epilepsy brain resections (patients 14, 15, 5, and 6). All images were acquired with a 20× objective. (B) Cells were scraped from fixed slides (patients 5 and 6), DNA was extracted, and DNA for HHV-6 U57 (major capsid protein) was detected by nested PCR. Negative and positive controls used were uninfected SupT1 T cells and HHV-6B (strain Z29)–infected SupT1 T cells, respectively. (36 KB JPG) [file pmed.0040180.sg001.jpg]
